# Genotype by environment interaction analysis for seed cotton yield stability under normal irrigation and drought stress conditions using numerical stability statistics

**DOI:** 10.1038/s41598-025-33327-6

**Published:** 2026-02-09

**Authors:** W. M. B. Yehia, Essam F. El-Hashash, Karima Mohamed El-Absy, Hassan A. Mesbah

**Affiliations:** 1 Cotton Research Institute, Agriculture Research Center, Giza, 12619 Egypt; 2 Agronomy Department, Faculty of Agriculture, Al-Azhar University, Cairo, 11651 Egypt; 3 Biology Department, University College of Tayma, University of Tabuk, P.O. Box 741, Tabuk, Saudi Arabia; 4 Agricultural Botany Department, Genetics Unit, Faculty of Agriculture, Al-Azhar University, Cairo, 11651 Egypt

**Keywords:** Stability statistics, Parametric, Non-parametric, AMMI, Drought, PCA, Seed cotton yield, Genetics, Plant sciences

## Abstract

One of the study’s main goals is to find high-yielding and stable genotypes in cotton under normal irrigation conditions (NIC) and drought stress conditions (DSC), as well as the comparison between parametric and non-parametric stability statistics. In order to achieve this objective, 24 cotton genotypes were evaluated under NIC and DSC during the 2019 and 2020 growing seasons (four environments) at the Sakha Agriculture Research Station in the Kafr El-Sheikh Governorate of Egypt. Every trial was set up using a randomized complete block design with three replications. According to the ANOVA, years under NIC and genotypes under DSC had a highly significant impact on the seed cotton yield. The AMMI analysis showed significant effects of environments (P < 0.01), genotypes, and their interaction (GEI) (P < 0.05) on seed cotton yield in four environments (two years and two irrigation conditions). The AMMI model successfully divided the variability by GEI into three principal component axes (PCs), only PC1 explained 87% of the total variability of GEI. When compared to NIC, seed cotton productivity across DSC was found to be significantly reduced, ranging from 8.54% (G5 genotype) to 42.38% (G3 genotype). The stability statistics have the ability to separate, rank, and detect stable genotypes in both irrigation conditions. According to *Y*_*i*_, *YS*_*i*_ and *TOP* stability measures, the genotypes G1, G5, G20 and G19 were determined as the most stable genotypes (dynamically) with the highest yielding performance, while, the genotypes G8 and G4 were the most stable (statically) with moderate seed cotton yield using most other stability statistics. Based on rank, Spearman’s rank correlation, PCA biplot and heatmap data, the stability parameters can be divided into two major groups that corresponded to different dynamic and static concepts of stability under NIC and DSC. The first group includes *YS*_*i*_ and *TOP*, which are strongly correlated with *Y*_*i*_ (dynamic). While, the second group has other stability statistics, which negatively correlated with *Y*_*i*_, thus related to the static concept. Generally, the genotypes G5 and G8 were stable coupled with high and moderate yield, respectively. Therefore, these genotypes are used in the cotton breeding programs for the development of improved cotton varieties to address drought conditions in Egypt.

**Supplementary Information:**

The online version contains supplementary material available at 10.1038/s41598-025-33327-6.

## Introduction

Cotton, a significant fiber crop, meets 35% of the global total fiber demands^1^. Cotton is one of the major important cash plants, play an important role in fiber and oil industrial development, as well as employment generation in Egypt. Cotton is subjected to drought and high temperatures because it is primarily grown in warmer climates^2^. El-Hashash and Agwa (2018)^3^ reported that all global crop species must investigate the potential of drought-tolerant crops in light of climate change. The expression of both drought avoidance (deep roots, leaf rolling, etc.) and tolerance (tissue tolerance, photosystem maintenance, etc.) traits is known as drought resistance, which is controlled by several genes^4^.

Increasing stability and stabilizing crop production during conditions (locations and/or years) is the primary goal of plant breeding programs^5^. When genotypes are evaluated different environments, their yield performances may vary considerably^6^. The genotype x environment interaction (GEI) is the term used to describe the relationship between a genotype’s phenotypic expression and its environment. In plant breeding programs, GEI is a regular occurrence or phenomenon. Scale disparities between environments, changes in the magnitude of genotype-to-environment variances, or fluctuations in the relative ranking of genotypes can all contribute to it^7–9^. One significant step toward the creation of improved plant genotypes is the assessment and comprehension of the GEI^10^. Breeders assess genotypes in many contexts (locations and/or years) to find those with high and consistent performance and higher adaptation to account for the effects of genetic engineering^11^. It is thought that genotypes with negligible GEI are stable^12^. For assessing and understanding GEI, biometricians can employ a variety of statistical modeling techniques, such as parametric and non-parametric stability statistics.

Numerous techniques employ GEI in conjunction with the genotypes’ mean yield as a selection index and to aid in genotype characterization^5^. Because of this, genotypes that exhibit little fluctuation in yield across environments, both high and low-yielding, are considered stable. This idea of stability can be viewed as either static or biological^13^. This idea of stability is rejected by the majority of plant crop breeders and agronomists, who instead favor genotypes with high average yields and the capacity to adapt to improved environmental circumstances or agronomic inputs. A dynamic (agronomical) approach of stability is preferred by breeders, who view excellent yield performance in released varieties as one of their primary objectives^14^. Genotype varies predictably under a variety of environmental circumstances in dynamic stability^13^. Numerous stability criteria that may be utilized to assess the stability of genotype performance have been created as a result of the occurrence of GE interaction. Huehn (1996)^15^ and Romagosa and Fox (1993)^16^ both state that parametric (empirical and statistical) and nonparametric (analytical clustering) methods are the two main ways to examine GE interaction to assess genotype adaptation. The reader might think that the word “stability” is a little ambiguous or subjective. It cannot be any other way, which is the primary cause of the wide variety of measurement techniques. They are all made to fill in the gaps that others have left^17^.

Based on statistical presumptions regarding the distribution of GEI effects, environment, and genotype, the parametric approach was developed. Plant breeders frequently employ parametric techniques to estimate phenotypic stability, and these techniques have been associated primarily with variance components and associated statistics^3^. The parametric methods range from univariate to multivariate techniques, for example, the methods proposed by Francis and Kannenberg’s (1978)^18^, Eberhart and Russell (1966)^19^, Wricke’s (1962)^20^, Hernandez et al. (1993)^21^, and Shukla’s (1972)^22^. Regarding the environment and phenotype of biotic and abiotic environmental elements, the nonparametric approach does not require any assumptions. The genotypes ranks in every environment are the basis for a number of nonparametric approaches proposed by Nassar and Huehn (1987)^23^, Kang’s (1988)^7^, Fox et al. (1990)^24^, and Thennarasu (1995)^25^, where the genotypes with similar rankings during environments are considered stable. While a number of models have been put out for the statistical measurement of stability, no single technique is sufficient to explain genotype performance in a given environment. However, for practical purposes, the majority of breeding programs currently include certain aspects of parametric and non-parametric techniques^14^.

Using parametric and non-parametric stability statistics, this study sought to 1) evaluate the stability ability of 24 cotton genotypes across NIC and DSC; 2) comprehend environmental, genotype, and interaction effects; and 3) explore correlations, similarities, and differences among these stability statistics. In order to ensure sustainable production in Egypt during drought conditions, these initiatives will aid in the selection of cotton genotypes with high yield, stability, and adaptability.

## Materials and methods

### Material and experimental procedure

The Sakha Agriculture Research Station in the Kafr El-Sheikh Governorate of Egypt is the site of the current study. Genotypes of 24 different genotypes of cotton (*Gossypium barbadense* L.) were selected (Table 1). In a randomized complete block design with three replications, 24 cotton genotypes were assessed in both NIC and DSC throughout the 2019 and 2020 growing seasons. Every genotype was sowed in an experimental plot consisting of five rows, each measuring four meters in length. The plant and row distances were maintained at 30 and 70 cm, respectively. The plot size kept was 13 m^2^. Eight irrigations (4200 m^3^) were applied under NIC: one at planting and seven more at intervals of 15 days at different phases of crop growth. The plot was irrigated five times (3150 m^3^) under DSC; the first irrigation was applied at the time of planting, and the remaining four were applied at 30-day intervals. In every experiment, a basin irrigation system with PE pipes and a volumetric counter was employed. In the drought stress studies, no further irrigation was given after drainage, even in cases when the DSC was severe. The crop was sown in a single day under uniform field conditions, adhering to all recommended cultural procedures for cotton production in the area, to minimize environmental fluctuations. Once the border effects were removed, the plants in every plot from the three center/middle rows were collected. This produced the seed cotton yield per plot in kg was transformed to kantars per feddan (1 Kentar = 157.5 kg).

**Table 1:** A list of 24 cotton genotypes investigated across NIC and DSC.

No	Genotypes	Pedigree	Origin
G1	Giza 89	Giza 89 × 6022	Egypt
G2	Z101	Unknown	Uzbekistan
G3	Giza 85	Giza 67 × CB58	Egypt
G4	Giza 75	Unknown	Egypt
G5	Giza 94	10,229 × Giza 86	Egypt
G6	A106	Unknown	Australia
G7	A101	Unknown	Australia
G8	Z102	Unknown	Uzbekistan
G9	Giza 89 × Giza 86	Unknown	Egypt
G10	Giza 45	Giza 28 × Giza 7	Egypt
G11	A108	Unknown	Australia
G12	Giza 93	Giza 77 × S106	Egypt
G13	D101	Unknown	India
G14	Giza 70	Giza 59 A x Giza 51B	Egypt
G15	A105	Unknown	Australia
G16	G102	Unknown	Greece
G17	R101	Unknown	Russian
G18	G101	Unknown	Greece
G19	Giza 96	(Giza 84 x (Giza 70 × Giza 51B)) x S62	Egypt
G20	Giza 86	Giza 75 × Giza 81	Egypt
G21	Giza 95	(Giza 83 x (Giza 75 × 5844)) x Giza 80	Egypt
G22	S106	Unknown	USA
G23	S107	Unknown	USA
G24	S109	Unknown	USA

#### Climatic data

**Fig. 1:**
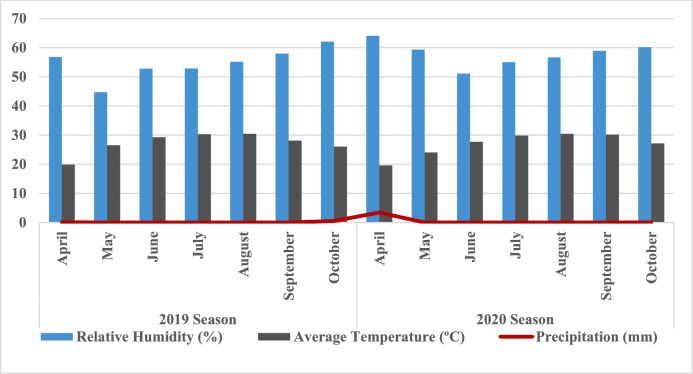
Monthly relative humidity, average temperature, and average monthly precipitation were collected during the 2019 and 2020 growing seasons at the experimental location, from April to October.

Climate data for cultivated location, including relative humidity (%), average temperature (ºC) and precipitation (mm), are shown in Fig. 1 for the two growth seasons spanning April through October. The Climate Change Information Center and Renewable Energy, Agriculture Research Center, Cairo, Egypt, provided these climate data. Higher temperatures were observed in August for both growing seasons. Still, they increased more in the 2019 growing season than in the 2020 growing season. Lower temperatures were found in April during both growing seasons. Generally, the 2019 growing season recorded the highest temperatures compared with the 2020 growing season. All months in the 2020 growing season were higher for average relative humidity than in the 2019 growing season, except June and October. The highest average relative humidity was noticed in October in both growing seasons. While lower average relative humidity was found in May and June during the 2019 and 2020 growing seasons, respectively. The highest values of total precipitation (mm) were recorded during October and April in the 2019 and 2020 growing seasons, respectively. Generally, total precipitation in the 2020 growing season was higher than in the 2019 growing season.

### Statistical analysis

The effects of years, genotypes, and their interaction on seed cotton yield across NIC and DSC were assessed using a combined ANOVA. The Additive Main Effects and Multiplicative Interaction Model (AMMI) was performed by Gauch (1988)^26^ to determine the main and interactions effects of genotypes and four environments (two years and two irrigation conditions) on seed cotton yield. F-tests were used to determine statistical tests of significance for these factors. To cover a broad spectrum of stability analysis approaches under NIC and DSC, sixteen parametric and non-parametric stability statistics were chosen. The parametric stability statistics were performed in accordance Francis and Kannenberg (1978)^18^ coefficient of variation (*CV*_*i*_), Eberhart and Russel’s (1966)^19^ linear regression coefficient (*b*_*i*_) and the variance of the regression deviations (${S}_{di}^{2}$), Wricks’s (1962)^20^ ecovalance (${W}_{i}^{2}$), Hernandez et al. (1993)^21^ desirability index (*D*_*i*_) and Shukla’s (1972)^22^ stability variance (${\sigma }_{i}^{2}$). The non-parametric statistical techniques selected for the stability analysis of the genotypes were *YS*_*i*_ by Kang (1988)^7^, ${S}_{i}^{(1)}$, ${S}_{i}^{(2)}$, ${S}_{i}^{(3)}$ and ${S}_{i}^{(6)}$ by Nassar and Huehn (1987)^23^, *TOP* by Fox et al. (1990)^24^, and ${NP}_{i}^{(1)}$, ${NP}_{i}^{(2)}$, ${NP}_{i}^{(3)}$ and ${NP}_{i}^{(4)}$ by Thennarasu’s (1995)^25^. Principal component analysis (PCA), Spearman’s rank correlation coefficients, and heatmap analysis were used to better understand the associations among all possible pair-wise comparisons of seed cotton yield and the stability methods. ANOVA, the AMMI model, parametric and non-parametric stability statistics, PCA, Spearman’s rank correlation coefficients, and heatmap analysis were conducted using the software program PBSTAT-GE 3.0.3 (Suwarno et al. 2025)^27^.

## Results

### Analysis of variance and AMMI model

**Fig. 2:**
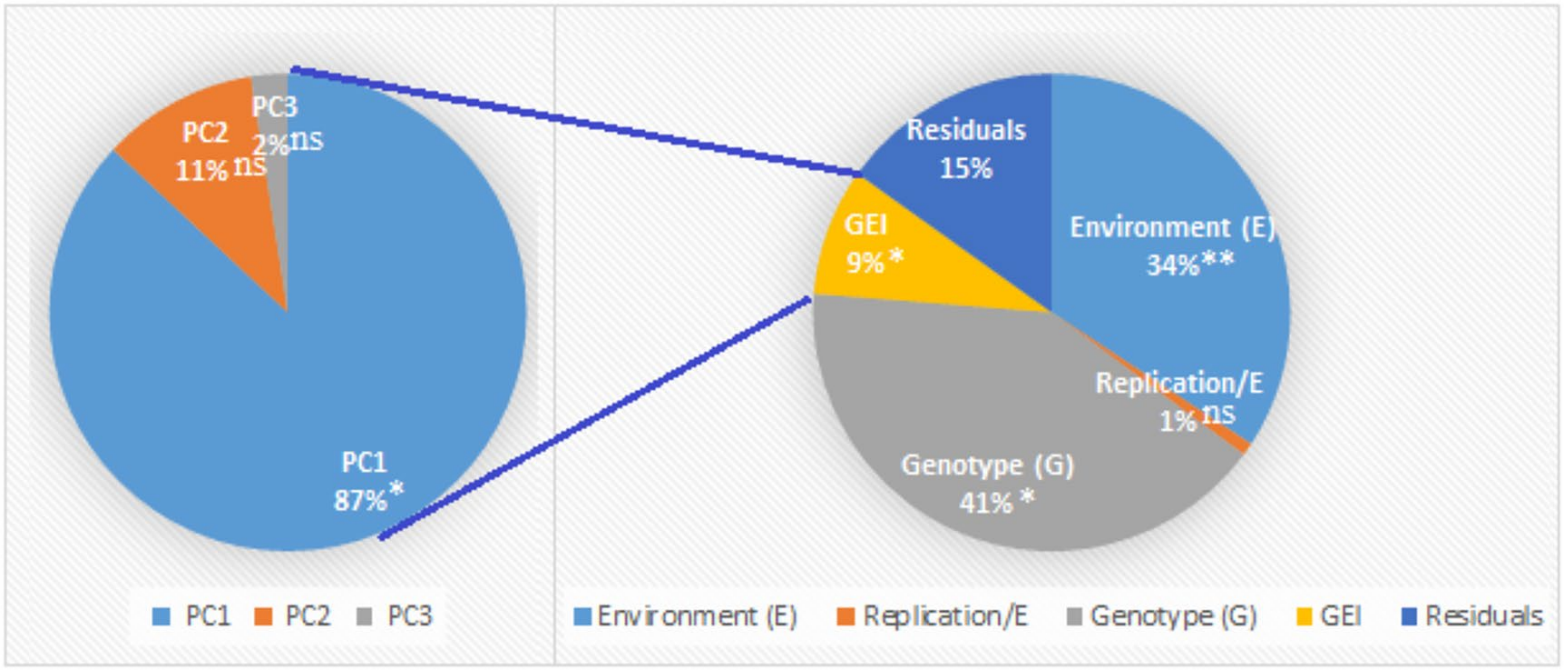
Combined ANOVA with AMMI analysis for seed cotton yield of 24 genotypes across four environments (two seasons and two irrigation conditions). *p ≤ 0.05 and **p < 0.01: statistically significant differences; ns: non-significant difference.

The analysis of variance (Table 2) showed highly significant between years (Y) under NIC, and between the 24 genotypes (G) in both conditions for seed cotton yield (kentars feddan^−1^). From the total sum of squares (SS), the SS for genotypes had the highest component across NIC and DSC with values of 71.27% and 73.20%, respectively. When under NIC and DSC, the seed cotton yield showed a moderate and high coefficient of variations (CV%) with values of 14.82% and16.87%, respectively. For the 24 cotton genotypes tested in four environments (two years and two irrigation conditions), the analysis of variance using the AMMI model revealed significant differences between environments (P < 0.01), genotypes (G), and GxE interaction (GEI) (P < 0.05) for seed cotton yield (Fig. 2). The GEI component was partitioned into three principal component axes (PCs). Only, the first principal component (PC1) was highly significant and obtained about 87% from the SS for GEI.

**Table 2:** Effect of genotype, year and their interaction on seed cotton yield using ANOVA of pooled data for studied genotypes during NIC and DSC over two growing seasons.

SOV	df	NIC			DSC		
		Sums of squares (SS)	Mean of squares	SS%	Sums of squares (SS)	Mean of squares	SS%
Years (Y)	1	28.94	28.94**	3.09	0.03	0.03^ ns^	0.01
Block within Y	4	15.37	3.84^ ns^	1.64	3.67	0.92^ ns^	0.69
Genotypes (G)	23	667.32	29.01**	71.27	387.45	16.85**	73.20
Y x G	23	19.31	0.84^ ns^	2.06	12.81	0.56^ ns^	2.42
Pooled Error	92	205.44	2.23	21.94	125.31	1.36	23.68
Total	143	936.38	6.55	100	529.27	3.70	100.00
CV%		14.82			16.87		

*p ≤ 0.05 and **p < 0.01: statistically significant differences; ns: non-significant difference.

### Genotypic mean performance

Genotypic Mean Performance: The data in Table 3 exhibited a highly significant variability in the genotype performances when comparing the means values with the values of LSD for seed cotton yield in both NIC and DSC. The results indicated that seed cotton yield had been impacted by DSC compared NIC, where drought stress caused a decrease in seed cotton yield of all genotypes studied. The reduction of seed cotton yield ranged from 8.54% in the G5 genotype to 42.38% in the G3 genotype. These results suggested that while certain genotypes behaved admirably, as demonstrated by a high ability to cotton yield even in a DSC, other genotypes were more negatively impacted by DSC. The 2019 year significantly increased seed cotton yield (2.18%) compared with the 2020 year under NIC. While under DSC, the effects of years were not significant. The G5 genotype had a maximum seed cotton yield (16.03 kentars feddan^−1^) in the 2019 year under NIC, while the minimum seed cotton yield (4.40 kentars feddan^−1^) was recorded by G12 genotype in the same year across DSC. In both years, the highest mean performances were observed by the genotypes G1, G5, G20 and G19 under both NIC and DSC, by G2 and G3 genotypes under NIC, and by G6 and G17 genotypes under DSC.

**Table 3:** Seed cotton yield (kentars feddan-1) of 24 selected genotypes across NIC and DSC during two growing years.

Genotypes	NIC			DSC		
	2019	2020	Mean	2019	2020	Mean
G1	12.68	15.37	14.03	10.33	10.62	10.48
G2	10.84	12.63	11.74	7.04	7.17	7.11
G3	13.20	13.43	13.32	6.30	4.48	5.39
G4	10.68	11.52	11.10	7.74	7.51	7.63
G5	16.03	15.33	15.68	9.96	8.94	9.45
G6	9.55	10.04	9.80	7.89	8.18	8.04
G7	8.08	9.40	8.74	5.30	5.26	5.28
G8	10.30	11.43	10.87	7.88	7.83	7.86
G9	9.67	10.29	9.98	7.43	7.40	7.42
G10	7.00	8.00	7.50	6.07	5.49	5.78
G11	8.69	9.26	8.98	6.04	5.30	5.67
G12	6.54	8.30	7.42	4.40	4.72	4.56
G13	7.66	8.02	7.84	6.03	5.79	5.91
G14	9.12	9.21	9.17	5.12	5.20	5.16
G15	8.60	9.19	8.90	7.30	7.69	7.50
G16	9.17	9.81	9.49	5.30	5.52	5.41
G17	9.65	9.80	9.73	7.94	8.10	8.02
G18	8.66	9.20	8.93	5.60	5.50	5.55
G19	10.69	13.08	11.89	8.75	9.35	9.05
G20	11.34	12.95	12.15	9.31	9.89	9.60
G21	9.30	10.15	9.73	7.21	8.21	7.71
G22	7.35	8.19	7.77	4.82	4.84	4.83
G23	6.38	7.33	6.86	4.97	5.69	.33
G24	10.04	10.82	10.43	7.00	7.71	7.36
Mean	9.63	10.53	10.08	6.91	6.93	6.92
LSD for genotypes	0.05	1.71		1.34		
	0.01	2.27		1.77		

### Stability statistics and their ranks

#### Parametric stability statistics

Various parametric and non-parametric stability statistics were utilized in stability analyses of seed cotton yield for 24 cotton genotypes under four environments (two years and two irrigation conditions), as shown in Table 4. The mean seed cotton yield of studied genotypes across NIC and DSC in both years ranged from 5.99 (kentars feddan^−1^) by the G12 genotype to 12.57 (kentars feddan^−1^) by the G5 genotype and the overall mean grain yield was 8.50 (kentars feddan^−1^). Thirteen genotypes performed better than the grand mean when the mean productivity was used as the first parameter for assessing the genotypes in both irrigation conditions.

**Table 4:** Mean response and stability statistics for seed cotton yield of 24 cotton genotypes tested in four environments.

Genotypes	Parametric stability statistics							Non-parametric stability statistics														
	*Y*_*i*_	${CV}_{i}$	${b}_{i}$	${S}_{di}^{2}$	${W}_{i}^{2}$	*D*_*i*_	${\sigma }_{i}^{2}$	*YS*_*i*_	*S*_*i*_^*(1)*^	*Z*_*i*_^*(1)*^	*S*_*i*_^*(2)*^	*Z*_*i*_^*(2)*^		*S*_*i*_^*(3)*^	*S*_*i*_^*(6)*^		*TOP*	*NP*_*i*_^*(1)*^	*NP*_*i*_^*(2)*^		*NP*_*i*_^*(3)*^	*NP*_*i*_^*(4)*^
G1	12.25	19.00	1.20^ ns^	0.09^ ns^	1.78	3.43	1.82	26 +	9.17	0.19	60.92	0.20		2.00	2.00		4.00	5.25	5.25		4.51	6.11
G2	9.42	29.41	1.48**	−0.54^ ns^	2.55	3.24	2.66	22 +	12.83	3.15	115.58	5.45		5.56	1.56		0.00	9.25	1.03		1.03	1.43
G3	9.35	49.55	2.42**	1.27*	24.59	3.76	26.7	10	15.33	7.23	176.33	19.64		30.02	3.07		2.00	11.50	1.35		1.07	1.43
G4	9.36	21.76	1.09^ ns^	−0.58^ ns^	0.13	3.23	0.02	19 +	5.83	0.62	21.58	0.83		1.30	0.67		0.00	3.75	0.50		0.49	0.71
G5	12.57	28.91	1.86**	1.15^ ns^	11.24	3.73	12.14	19 +	13.67	4.32	134.00	8.83		2.11	1.56		3.00	10.00	5.00		4.46	6.07
G6	8.91	11.71	0.56**	−0.58^ ns^	2.08	3.23	2.14	17 +	10.83	1.09	76.25	0.96		4.00	1.33		0.00	7.25	0.81		0.84	1.20
G7	7.01	29.55	1.11^ ns^	−0.57^ ns^	0.18	3.24	0.08	4	6.50	0.30	26.92	0.53		0.92	0.38		0.00	4.25	0.22		0.24	0.35
G8	9.36	19.20	0.96^ ns^	−0.58^ ns^	0.05	3.23	−0.07	20 +	2.33	4.28	3.33	2.37		0.10	0.19		0.00	1.50	0.19		0.20	0.30
G9	8.70	17.25	0.81^ ns^	−0.60^ ns^	0.40	3.23	0.32	13 +	5.00	1.19	16.67	1.16		0.46	0.34		0.00	2.50	0.25		0.34	0.49
G10	6.64	16.52	0.56**	−0.45^ ns^	2.27	3.27	2.35	1	12.17	2.34	92.25	2.34		2.10	0.62		0.00	7.75	0.39		0.43	0.62
G11	7.32	26.59	1.03^ ns^	−0.42^ ns^	0.36	3.28	0.27	7	8.00	0.00	41.67	0.05		0.40	0.27		0.00	5.00	0.31		0.33	0.48
G12	5.99	30.17	0.95^ ns^	−0.36^ ns^	0.51	3.30	0.43	−2	5.33	0.94	17.67	1.09		0.40	0.22		0.00	3.00	0.13		0.16	0.24
G13	6.88	16.39	0.60**	−0.57^ ns^	1.71	3.23	1.74	3	10.67	0.96	68.67	0.51		2.01	0.60		0.00	6.50	0.35		0.39	0.58
G14	7.16	32.26	1.22^ ns^	−0.33^ ns^	1.03	3.31	10.00	5	9.83	0.46	65.58	0.37		1.46	0.54		0.00	6.25	0.33		0.38	0.53
G15	8.19	10.46	0.45**	−0.56^ ns^	3.22	3.24	3.39	10	13.50	4.07	127.58	7.56		5.11	1.19		0.00	9.75	0.70		0.69	0.95
G16	7.45	31.82	1.27^ ns^	−0.52^ ns^	0.89	3.25	0.85	8	10.50	0.85	72.25	0.71		1.35	0.52		0.00	7.25	0.48		0.47	0.68
G17	8.87	11.11	0.52**	−0.57^ ns^	2.43	3.24	2.52	15 +	11.67	1.81	88.67	1.98		5.27	1.41		0.00	7.50	0.83		0.88	1.26
G18	7.24	27.13	1.05^ ns^	−0.55^ ns^	0.12	3.24	0.00	6	5.33	0.94	20.33	0.91		0.06	0.11		0.00	3.00	0.17		0.22	0.30
G19	10.47	18.36	0.97^ ns^	0.09^ ns^	1.38	3.43	1.39	24 +	11.00	1.22	75.00	0.87		1.12	0.82		1.00	7.00	1.75		1.76	2.59
G20	10.87	14.99	0.84^ ns^	−0.33^ ns^	0.78	3.31	0.73	25 +	6.83	0.18	29.58	0.40		1.43	1.14		2.00	3.75	1.07		1.35	1.95
G21	8.71	14.70	0.65*	−0.34^ ns^	1.79	3.31	1.83	14 +	8.50	0.04	47.58	0.00		3.60	1.00		0.00	5.25	0.48		0.60	0.85
G22	6.30	27.43	0.93^ ns^	−0.60^ ns^	0.05	3.23	−0.06	0	3.83	2.31	8.92	1.81		0.13	0.14		0.00	2.25	0.10		0.12	0.18
G23	6.09	16.50	0.51**	−0.41^ ns^	2.92	3.28	3.06	−1	13.33	3.83	113.67	5.15		2.58	0.59		0.00	9.00	0.39		0.43	0.63
G24	8.89	20.54	0.97^ ns^	−0.48^ ns^	0.24	3.26	0.14	16 +	5.33	0.94	18.00	1.07		1.20	0.60		0.00	3.50	0.39		0.37	0.53
Grand mean		Sum(*Z*_*i*_^*(1)*^)		Sum(*Z*_*i*_^*(2)*^)		E(*S*_*i*_^*(1)*^)		E(*S*_*i*_^*(2)*^)		Var(*S*_*i*_^*(1)*^)		Var(*S*_*i*_^*(2)*^)			*x*^*2*^ table for *Z*_*i*_^*(1)*^, *Z*_*i*_^*(2)*^				*x*^*2*^ table for Sum(*Z*_*i*_^*(1)*^), (*Z*_*i*_^*(2)*^)			
8.50		43.23		64.78		7.99		47.92		7.47		839.47			9.47				36.42			

*Y*_*i*_: Mean response;${{\boldsymbol{C}}{\boldsymbol{V}}}_{{\boldsymbol{i}}}$: Francis and Kannenberg (1978)^18^ coefficient of variation; ${{\boldsymbol{b}}}_{{\boldsymbol{i}}}$ and ${{\boldsymbol{S}}}_{{\boldsymbol{d}}{\boldsymbol{i}}}^{2}$ : Eberhart and Russel’s (1966)^19^ linear regression coefficient and the variance of the regression deviations, respectively; ${{\boldsymbol{W}}}_{{\boldsymbol{i}}}^{2}$: Wricks’s (1962)^20^ ecovalance; ${{\boldsymbol{D}}}_{{\boldsymbol{i}}}$: Hernandez et al. (1993)^21^ desirability index; ${{\boldsymbol{\sigma}}}_{{\boldsymbol{i}}}^{2}$: Shukla’s (1972)^22^ stability variance; *YS*_*i*_: Kang’s yield and stability index; *S*_*i*_^*(1)*^, *S*_*i*_^*(2)*^, *S*_*i*_^*(3)*^, *S*_*i*_^*(6)*^: Nassar and Huehn (1987)^23^ nonparametric stability parameters; *Z*_*i*_^*(1)*^, *Z*_*i*_^*(2)*^: the Z-statistics are measures of stability for *S*_*i*_^*(1)*^ and *S*_*i*_^*(2)*^; *TOP*: Fox et al. (1990)^24^ number of sites at which the genotype occurred in the top third of ranks; *NP*_*i*_^*(1)*^, *NP*_*i*_^*(2)*^, *NP*_*i*_^*(3)*^, *NP*_*i*_^*(4)*^: Thennarasu’s (1995)^25^ nonparametric stability parameters.

The highest values of seed cotton yield (*Y*_*i*_) and desirability index (*D*_*i*_) indicate that a genotype’s performance was more stable under environments, the opposite is true for ${CV}_{i}$, ${W}_{i}^{2}$, and ${\sigma }_{i}^{2}$ statistics. The genotypes G1, G5, G20, and G19 by *Y*_*i*_ statistic registered the maximum values and represented the most stable genotypes, unlike, the genotypes G12, G22 and G23 under NIC and DSC. While, according to the *D*_*i*_ statistic, the genotypes G22, G9 and G6 registered the maximum *D*_*i*_ values and thus were stable, but these genotypes had low to moderate cotton yield.

The results of the Eberhart and Russell model including regression coefficients (${b}_{i}$) and deviation from regression $({S}_{di}^{2})$ indicate that genotypes already responded to both irrigation conditions changes differently. Ten genotypes including G2, G3, G5, G6, G10, G13, G15, G17, G1 and G23 had significant ${b}_{i}$ values different from one, while, G3 genotype had a significant ${S}_{di}^{2}$ value higher than zero, only. The genotype G11 (${b}_{i}$>1) and the genotype es G24, G19, G8, G18 (${b}_{i}$<1) were close to the unity of ${b}_{i}$, while the genotypes G22, G9, G6, G8 and G4 had a deviation different from zero. According to the ${b}_{i}$ and ${S}_{di}^{2}$ these genotypes show specific adaptability in environments and with moderate cotton yield performance. G19 genotype with the high seed cotton yield ranked 3^rd^ and 21^th^ by ${b}_{i}$ and ${S}_{di}^{2}$, respectively. This genotype exhibited stability and yield in an excellent ratio. G5, G1, G20 and G2 genotypes could be low stable or unstable based on ${b}_{i}$ and ${S}_{di}^{2}$ parameters, but they registered the greatest seed cotton yield under NIC and DSC.

Regarding ${CV}_{i}$, the genotypes G15, G17, G6, G21and G20 were more stable. Only G20 had high seed cotton yield, while other genotypes were moderate to low productivity performance. On the other side, the ${W}_{i}^{2}$ and ${\sigma }_{i}^{2}$ parameters produced the lowest values for genotypes G8, G22, G18, G4, and G7, with the best seed cotton yield only for the G8 and G4 genotypes. Because these genotypes contributed the least to the GEI based on ${W}_{i}^{2}$ and ${\sigma }_{i}^{2}$ parameters, therefore, they were considered more stable than other studied genotypes.

#### Non-parametric stability statistics

A genotype’s performance was more consistent across environments when its *TOP* and Kang’s yield and stability index (*YS*_*i*_) values were at their highest. While, the genotypes with the lowest values of ${S}_{i}^{(1)}$, ${S}_{i}^{(2)}$, ${S}_{i}^{(3)}$, ${S}_{i}^{(6)}$, ${NP}_{i}^{(1)}$, ${NP}_{i}^{(2)}$, ${NP}_{i}^{(3)}$ and ${NP}_{i}^{(4)}$ performed better and more stable than the other genotypes under NIC and DSC. In contrast to the genotypes G12, G22, and G23 under NIC and DSC, the genotypes G1, G20, and G19 by *YS*_*i*_ and *TOP* statistics, as well as the G5 genotype by *TOP* statistics, were registered with the highest values and represented the most stable genotypes.

Nassar and Huehn (1987)^23^ state that ${Z}_{i}^{(1)}$ and ${Z}_{i}^{(2)}$ values determined based on the rank of the corrected data and added across genotypes to obtain the two overall chi-square stabilities, which are Sum(${Z}_{i}^{(1)}$) = 43.23 and Sum(${Z}_{i}^{(2)}$) = 64.78. The two overall chi-square stabilities were larger than the tabulated chi-square at the degree of freedom = 24, where 0.05 = 36.42 and 0.01 = 42.98, providing strong evidence for extremely significant differences in stability across the 24 cotton genotypes under NIC and DSC. Based on Nassar and Huehn (1987)^23^ and Thennarasu’s (1995)^25^ nonparametric stability parameters, the genotypes G7, G8, G9, G12, G18, and G22 recorded the lower values, thus genotypes are considered stable based on these parameters in comparison to the other genotypes. The lowest values were observed for G8 genotype (moderate yield) by ${S}_{i}^{(1)}$, ${S}_{i}^{(2)}$ and ${NP}_{i}^{(1)}$ statistics, for G18 genotype (low to moderate yield) by ${S}_{i}^{(3)}$ and ${S}_{i}^{(6)}$ statistics, and for G22 genotype (low yield) by ${NP}_{i}^{(2)}$, ${NP}_{i}^{(3)}$ and ${NP}_{i}^{(4)}$ statistics. Conversely, the two genotypes G3 and G5 were unstable based on the most stability parameters studied, however, the G5 genotype has a high cotton yield across NIC and DSC.

#### Stability statistics ranks

The ranks of 24 cotton genotypes using the parametric and non-parametric stability statistics are shown in Table 5. The pairs of stability parameters including ${S}_{di}^{2}$ and *D*_*i*_; ${W}_{i}^{2}$ and ${\sigma }_{i}^{2}$; ${S}_{i}^{(1)}$ and ${S}_{i}^{(2)}$; ${S}_{i}^{(2)}$ and ${NP}_{i}^{(2)}$ as well as ${NP}_{i}^{(3)}$ and ${NP}_{i}^{(4)}$ were identical in the rank of the studied genotypes. Additionally, most stability parameters showed similarly ranks of genotypes, for example *Y*_*i*_ and *YS*_*i*_; ${W}_{i}^{2}$, ${\sigma }_{i}^{2}$, ${S}_{i}^{(1)}$, ${S}_{i}^{(2)}$, ${S}_{i}^{(3)}$ and ${NP}_{i}^{(1)}$; as well as ${S}_{i}^{(6)}$, ${NP}_{i}^{(1)}$, ${NP}_{i}^{(2)}$, ${NP}_{i}^{(3)}$ and ${NP}_{i}^{(4)}$.

**Table 5:** Ranks of 24 cotton genotypes using stability parameters across four environments.

Genotypes	Parametric stability statistics							Non-parametric stability statistics									
	*Y*_*i*_	${CV}_{i}$	${b}_{i}$	${S}_{di}^{2}$	${W}_{i}^{2}$	*D*_*i*_	${\sigma }_{i}^{2}$	*YS*_*i*_	*S*_*i*_^*(1)*^	*S*_*i*_^*(2)*^	*S*_*i*_^*(3)*^	*S*_*i*_^*(6)*^	*TOP*	*NP*_*i*_^*(1)*^	*NP*_*i*_^*(2)*^	*NP*_*i*_^*(3)*^	*NP*_*i*_^*(4)*^
G1	2	11	12	22	15	22	15	1	12	12	14	23	1	11.5	24	24	24
G2	5	19	20	11	20	11	20	4	20	21	23	21.5	15	21	19	19	19
G3	8	24	24	24	24	24	24	13.5	24	24	24	24	3.5	24	21	20	20
G4	7	14	8	5	4	5	4	6.5	7	7	10	14	15	7.5	15	14	14
G5	1	18	23	23	23	23	23	6.5	23	23	17	21.5	2	23	23	23	23
G6	9	3	18	3	17	3	17	8	16	17	20	19	15	16.5	17	17	17
G7	19	20	9	7	5	7	5	19	8	8	7	7	15	9	5	5	5
G8	6	12	4	4	1	4	1	5	1	1	2	3	15	1	4	3	3
G9	13	9	11	2	8	2	8	12	3	3	6	6	15	3	6	7	7
G10	21	8	17	14	18	14	18	21	19	19	16	13	15	19	10	11	11
G11	16	15	1	15	7	15	7	16	10	10	5	5	15	10	7	6	6
G12	24	21	6	17	9	17	9	24	5	4	4	4	15	4.5	2	2	2
G13	20	6	16	6	14	6	14	20	15	14	15	12	15	14	9	10	10
G14	18	23	13	19	12	19	12	18	13	13	13	9	15	13	8	9	8
G15	14	1	22	9	22	9	22	13.5	22	22	21	18	15	22	16	16	16
G16	15	22	14	12	11	12	11	15	14	15	11	8	15	16.5	14	13	13
G17	11	2	19	8	19	8	19	10	18	18	22	20	15	18	18	18	18
G18	17	16	5	10	3	10	3	17	5	6	1	1	15	4.5	3	4	4
G19	4	10	3	21	13	21	13	3	17	16	8	15	5	15	22	22	22
G20	3	5	10	20	10	20	10	2	9	9	12	17	3.5	7.5	20	21	21
G21	12	4	15	18	16	18	16	11	11	11	19	16	15	11.5	13	15	15
G22	22	17	7	1	2	1	2	22	2	2	3	2	15	2	1	1	1
G23	23	7	21	16	21	16	21	23	21	20	18	10	15	20	12	12	12
G24	10	13	2	13	6	13	6	9	5	5	9	11	15	6	11	8	9

### Spearman’s rank correlation coefficients

Table 6 displays the derived Spearman’s rank correlation coefficients for every pair of calculated statistics on seed cotton yield. A perfect correlation (r = 1.00) were noticed between ${S}_{di}^{2}$ and *D*_*i*_, between ${W}_{i}^{2}$ and ${\sigma }_{i}^{2}$, and among ${S}_{i}^{(1)}$, ${S}_{i}^{(2)}$, and ${NP}_{i}^{(1)}$. Highly significant and positive association were noticed among *Y*_*i*_, *YS*_*i*_, and *TOP*, as well as among *b*_*i*_, ${W}_{i}^{2}$, ${\sigma }_{i}^{2}$, ${S}_{i}^{(1)}$, ${S}_{i}^{(2)}$, ${S}_{i}^{(3)}$, ${S}_{i}^{(6)}$, ${NP}_{i}^{(1)}$, ${NP}_{i}^{(2)}$, ${NP}_{i}^{(3)}$ and ${NP}_{i}^{(4)}$. The *Y*_*i*_ had positively correlated with *CV*_*i*_ and it negatively correlated with other parametric and non-parametric stability statistics (significant or insignificant). In the positive direction, the ${S}_{di}^{2}$ and *D*_*i*_ were significantly associated with ${W}_{i}^{2}$, ${\sigma }_{i}^{2}$, ${S}_{i}^{(1)}$, ${S}_{i}^{(2)}$,${S}_{i}^{(6)}$, ${NP}_{i}^{(1)}$ (P < 0.05), ${NP}_{i}^{(2)}$, ${NP}_{i}^{(3)}$ and ${NP}_{i}^{(4)}$ (P < 0.01).

**Table 6:** Spearman’s rank correlation coefficients among seed cotton yield and stability statistics ranks for 24 genotypes across four environments.

Statistics	Parametric stability							Non-parametric stability								
	*Y*_*i*_	${CV}_{i}$	${b}_{i}$	${S}_{di}^{2}$	${W}_{i}^{2}$	*D*_*i*_	${\sigma }_{i}^{2}$	*YS*_*i*_	*S*_*i*_^*(1)*^	*S*_*i*_^*(2)*^	*S*_*i*_^*(3)*^	*S*_*i*_^*(6)*^	*TOP*	*NP*_*i*_^*(1)*^	*NP*_*i*_^*(2)*^	*NP*_*i*_^*(3)*^
${CV}_{i}$	0.11^ ns^															
${b}_{i}$	−0.08^ ns^	−0.18^ ns^														
${S}_{di}^{2}$	−0.30^ ns^	0.24^ ns^	0.18^ ns^													
${W}_{i}^{2}$	−0.19^ ns^	−0.24^ ns^	0.88**	0.47*												
*D*_*i*_	−0.30^ ns^	0.24^ ns^	0.18^ ns^	1.00**	0.47*											
*YS*_*i*_	−0.19^ ns^	−0.24^ ns^	0.88**	0.47*	1.00**	0.47*										
${\sigma }_{i}^{2}$	0.97**	0.22^ ns^	0.03^ ns^	−0.19^ ns^	−0.09^ ns^	−0.19^ ns^	−0.09^ ns^									
*S*_*i*_^*(1)*^	−0.18^ ns^	−0.14^ ns^	0.82**	0.46*	0.94**	0.46*	0.94**	−0.06^ ns^								
*S*_*i*_^*(2)*^	−0.20^ ns^	−0.12^ ns^	0.83**	0.43*	0.93**	0.43*	0.93**	−0.08^ ns^	1.00**							
*S*_*i*_^*(3)*^	−0.26^ ns^	−0.30^ ns^	0.88**	0.28^ ns^	0.91**	0.28^ ns^	0.91**	−0.19^ ns^	0.85**	0.86**						
*S*_*i*_^*(6)*^	−0.64**	−0.26^ ns^	0.67**	0.44*	0.79**	0.44*	0.79**	−0.57**	0.74**	0.74**	0.86**					
*TOP*	0.66**	−0.09^ ns^	−0.17^ ns^	−0.70**	−0.34^ ns^	−0.70**	−0.34^ ns^	0.54**	−0.33^ ns^	−0.31^ ns^	−0.20^ ns^	−0.58**				
*NP*_*i*_^*(1)*^	−0.17^ ns^	−0.09^ ns^	0.83**	0.42*	0.93**	0.42*	0.93**	−0.05^ ns^	0.99**	1.00**	0.86**	0.73**	−0.27^ ns^			
*NP*_*i*_^*(2)*^	−0.76**	−0.22^ ns^	0.51*	0.53**	0.69**	0.53**	0.69**	−0.70**	0.69**	0.70**	0.69**	0.92**	−0.71**	0.67**		
*NP*_*i*_^*(3)*^	−0.73**	−0.27^ ns^	0.55**	0.54**	0.71**	0.54**	0.71**	−0.67**	0.70**	0.71**	0.71**	0.93**	−0.71**	0.68**	0.99**	
*NP*_*i*_^*(4)*^	−0.74**	−0.27^ ns^	0.54**	0.53**	0.71**	0.53**	0.71**	−0.68**	0.70**	0.70**	0.71**	0.93**	−0.71**	0.67**	0.99**	1.00**

*p ≤ 0.05 and **p < 0.01: statistically significant differences; ns: non-significant difference.

### PCA biplot of genotypes and stability parameters

**Fig. 3:**
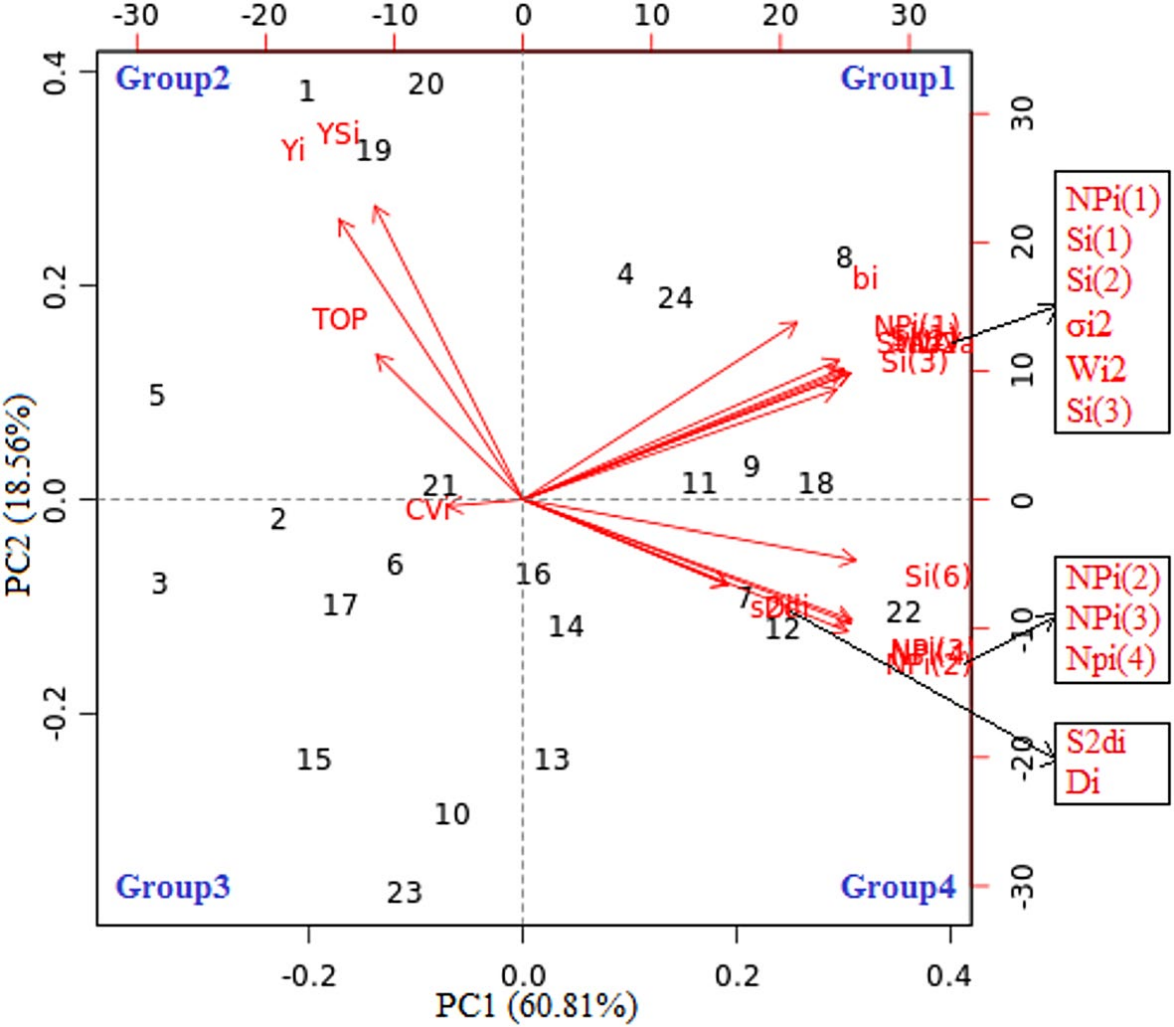
PCA biplot of genotypes and stability statistics based on PC1 and PC2 axes for seed cotton yield across four environments.

Based on their data from the NIC and DSC over the two years, the PC1 and PC2 were used to draw a biplot for the genotypes and stability parameters (Fig. 3). PC1 only described roughly 60.81% of the measured data total variability, its contributions to the total variance were greater than those of PC2 (18.56%). PC1 and PC2 account for 79.37%. Sharp, obtuse, and right angles indicate positive, negative correlation and no correlation between the stability parameters, respectively. The results of PCA analysis confirmed Spearman’s rank correlation coefficient results among stability parameters investigated across NIC and DSC. According to stability parameters studied (Fig. 3), the 24 cotton genotypes could also be categorized into four groups using PCA analysis for seed cotton yield under NIC and DSC. The genotypes G4, G8, G9, G11, G18, and G24 with the parameters *b*_*i*_, ${W}_{i}^{2}$, ${\sigma }_{i}^{2}$, ${S}_{i}^{(1)}$, ${S}_{i}^{(2)}$, ${S}_{i}^{(3)}$ and ${NP}_{i}^{(1)}$, made up the first group (the highest PC1 and PC2) and which demonstrated moderate seed cotton production under both irrigation conditions. The second group (the lowest PC1 and the highest PC2) was comprised of the genotypes G1, G5, G19, G20, and G21 using the stability parameters *Y*_*i*_, *YS*_*i*_, and *TOP*, which registered the highest seed cotton yield in NIC and DSC, except G21 genotype. The third group was composed of the genotypes G2, G3, G6, G10, G15, G17, and G23, which exhibited low to moderate seed cotton yield performance in both NIC and DSC, and were linked to the *CV*_*i*_ parameter. The fourth group, which included the other genotypes and other stability indices with the greatest PC1 and lowest PC2, showed poor to moderate seed cotton yield performance across NIC and DSC.

### Heatmap of genotypes and stability statistics

**Fig. 4:**
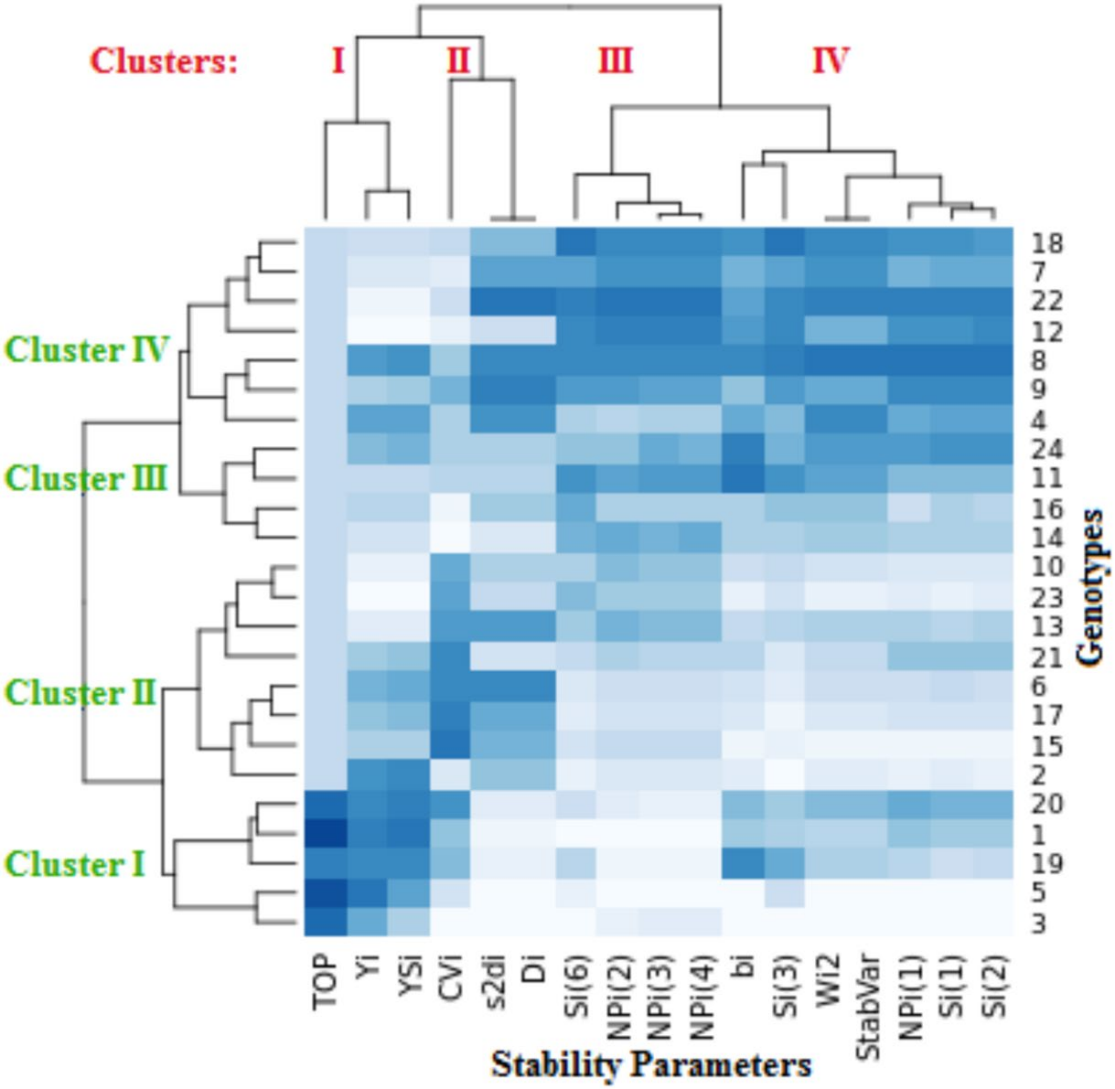
Genotypes and stability parameters Heatmap for seed cotton yield across four environments.

A visual comparison of the impacts of NIC and DSC during the two years on the genotypes based on parametric and non-parametric stability parameters was created using a heatmap analysis (Fig. 4). The heatmap analysis on the basis of stability parameters classified the genotypes into four clusters (green color). According to the dendrogram, 21%, 33%, 17% and 29% of genotypes were located in the first, second, third and fourth clusters in both NIC and DSC, respectively. The genotypes G1, G3, G5, G19 and G20 in the first cluster had the best cotton yield in both conditions and the most stable using *Y*_*i*_, *YS*_*i*_ and *TOP* parameters. These stability statistics as parameters of genotypic performance are attempting to combine both productivity and adaptability. These parameters were strongly positively associated with one another (Table 6), thus they could be viewed as suitable substitutes for one another because of this. The second cluster comprised the G2 genotype by *Y*_*i*_ and *YS*_*i*_ parameters, and the genotypes G6, G10, G13, G15, G17, G21 and G23 by *CV*_*i*_, ${S}_{di}^{2}$ and *D*_*i*_ parameters. While the third and fourth clusters consisted of the other genotypes using the other stability parameters measured. The genotypes in the second, third and fourth clusters recorded low to moderate seed cotton yield in NIC and DSC. In Fig. 4, the cluster analysis based on seed cotton yield for 24 cotton genotypes under NIC and DSC during the two years were carried out to determine the correlations between the parametric and non-parametric stability statistics. The estimations of stability statistics were divided into four clusters (red color). The first cluster (I) consisted of *Yi*, *YSi* and *TOP* parameters. The second cluster (III) have three parameters including *CV*_*i*_, ${S}_{di}^{2}$ and *D*_*i*_. The third cluster (III) contained parameters of ${S}_{i}^{(6)}$, ${NP}_{i}^{(2)}$, ${NP}_{i}^{(3)}$ and ${NP}_{i}^{(4)}$. The fourth cluster (IV) consists of remaining parameters.

## Discussion

Parametric and non-parametric stability parameters identify highly productive and stable genotypes in NIC and DSC. Given that different genotypes exhibit varying degrees of performance in the various environments, there is a high likelihood of significant GEI occurring in numerous settings. This phenomenon is well-known to the majority of researchers^28^. The ANOVA indicated that there were substantial differences in genotypic response under these environmental effects (NIC and DSC). Similar to Yehia (2020)^29^, Sun et al. (2021)^30^, Abo Sen et al. (2022)^31^, Rizwan et al. (2022)^32^, Yehia et al. (2023)^33^, and Deshmukh et al. (2025)^34^ discovered high variability in yield among cotton genotypes across NIC and DSC, allowing us to select genotypes under DSC in our study. Significant contributions from the environment, genotype, and GEI accounted for 79.58%, 3.2%, and 15.46% of the variation, respectively^35^. The size of CV% revealed that the genotypes had exploitable genetic variability during the selection of cotton output under DSC. Based on CV% values, the environment affects seed cotton production in both irrigation conditions. Yehia and El-Hashash (2021)^36^; El-Hashash and Yehia (2021)^37^ reported the CV% was less than 10%, while it was higher than 10% by Li et al. (2020)^38^.

AMMI model results indicated the environments studied were diverse and the cotton genotypes gave a distinguished performance in response to environmental variability. Our findings showed that the AMMI model successfully divided the variability in seed cotton yield, which can aid in the choice of the best genotypes under NIC and DSC. Using AMMI analysis of variance, Iqbal et al. (2022)^39^, Lingaiah et al. (2020)^40^, Maleia et al. (2019)^41^, Riaz et al. (2019)^42^, Yehia et al. (2023)^33^, and Yue et al. (2025)^43^ have previously identified significant interactions between genotypes, environments, and GEI on cotton quantitative traits. The presence of variations among genotypes justifies the use of stability statistics methods to identify stable genotypes with superior yield across various environmental conditions (El-Hashash et al. 2019)^44^.

Cotton genotypes under NIC conditions are high-yielding compared with DSC. These results align with the conclusions of other studies, such as AbdElgalil et al. (2025)^45^, Ayele et al. (2020)^46^, Abo Sen et al. (2022)^31^, Eid et al. (2022)^47^, Eissa et al. (2023)^48^, and Hawash et al. (2022)^49^. G1, G5, G20, and G19 cotton genotypes are high-yielding genotypes with above the grand mean seed cotton yield in both NIC and DSC. Therefore, a reduced yield reduction value, which is thought to be a crucial indicator for cotton stability, demonstrates a genotype’s capacity to function effectively under DSC. Yield stability is due to consistent results throughout a range of sites and/or years^50^. This variation in genotype yield across environments demonstrated that the GEI effect was a type of crossover^11^.

Marco et al. (2022)^51^, Murthy and Pradeep (2022)^52^, Sirisha et al. (2019)^53^, and Vavdiya et al. (2021)^54^ had previously reported on the stability of genotypes for seed cotton production. Generally, the genotypes G1, G5, G20 and G19 genotypes had the most stable and higher seed yield values than other genotypes using *Y*_*i*_, *YS*_*i*_ and *TOP* stability statistics. These stability parameters were related to dynamic stability, which is associated with the high productivity of cotton genotypes. *YS*_*i*_ might be one of the crucial factors in choosing the right genotypes, which concurs with the findings of Baraki et al. (2024)^35^. A genotype varies predictably over a variety of environmental conditions in dynamic stability^14^. Unlike previously stability statistics, the other statistics determined the genotypes which have moderate and low seed cotton yield, for example, G8 and G22 genotypes, respectively. These stability parameters are associated with static stability. The majority of plant breeders and agronomists reject the static idea of stability because they favor genotypes with high mean yields and the capacity to adapt to agronomic inputs or better environmental conditions^3^. In similar results, El-Hashash et al. (2019)^44^ reported that the most stable genotypes based on most non-parametric parameters had the lowest productivity, unlike the other genotypes. They added that it is possible to choose stable genotypes with a low mean yield based on low values of these parameters. Because of this, identifying stable genotypes with high productivity is difficult using these statistics^55^. Barroso et al. (2019)^56^ and Nascimento et al. (2021)^57^ showed that the quantile regression method provides superior results compared to the Eberhart and Russell model in the presence of influential points. Our results indicated that static stability focuses on performance consistency in stable environments, while dynamic stability considers adaptability to changing and diverse conditions. Both are important for cotton breeding and variety selection under drought conditions.

Similarly, Deshmukh et al. (2025)^34^ and Sadabadi et al., (2018)^58^ in cotton, Herawati et al., (2021)^59^ in rice, Al-Ashkar et al. (2023)^60^ in wheat and Shojaei et al. (2022)^61^ in maize, some stability statistics in this study were identical in the rank of the studied genotypes, according to stability parameters rank. These results indicate that these stability statistics are equal for selecting genotypes across NIC and DSC, therefore it is it is could be considered as suitable alternatives for each other and it is sufficient to use one of them, as reported by El-Hashash and Agwa (2018)^3^ and El-Hashash et al. (2019)^44^ for parametric and non-parametric stability statistics, respectively. El-Hashash et al. (2019)^44^ reported that a single statistic could not determine stable genotypes and that non-parametric stability parameters differed in determining stable genotypes. For example, in our study, the genotypes G5, G1, G20, and G19 occupied the first four ranks using statistical measures *Y*_*i*_, *YS*_*i*_ and *TOP*, thus they had the most stable, while they had unstable by other statistical measures. Whilst the exact opposite was observed for genotypes G8, G22, G18 and G9. The yield rank and variance rank of every genotype are combined, and the resulting number is used as a statistic to determine whether genotype is stable^61^.

Spearman’s rank correlation coefficient and PCA analysis were utilized to comprehend the relationships among the stability statistics studied. Our results showed a perfect correlation among some stability statistics and highly significant and positive associations among *Yi*, *YSi*, and *TOP* were found. The complete association between stability parameters indicated their close similarity and effectiveness in ranking genotypes for stability under different environments^44^. Consequently, these stability statistics shouldn’t be treated as separate procedures^62^. On the other hand, positive and negative correlations were noticed in this study. These results coincide with that reported by El-Hashash and Agwa, (2018)^3^, El-Hashash et al. (2019)^44^, Herawati et al. (2021)^59^, Khan et al. (2020)^63^, and Shojaei et al. (2022)^61^ in barley, soybean, wheat, rice and maize, respectively. The *Y*_*i*_ displayed a negative association with ${NP}_{i}^{(2)}$, ${NP}_{i}^{(3)}$ and ${NP}_{i}^{(4)}$, while a positive association with *YS*_*i*_, ${S}_{i}^{(1)}$, ${S}_{i}^{(3)}$, ${S}_{i}^{(6)}$, according to Baraki et al. (2024)^35^. The strong relationship among *YS*_*i*_ and *TOP* with the highest productivity, as well as among other stability parameters studied with moderate or low productivity suggests that these statistics would play similar roles in the stability ranking of genotypes. As a result, any one of them can be utilized to choose stable and high-yielding genotypes of cotton. According to the non-significant correlation between mean yield and stability statistics, these statistics offer information that cannot be obtained from the mean yield alone^64^. On the contrary, then these statistics offer data that can be obtained from the mean yield alone^3^. Significant positive Spearman’s rank correlation of stability parameters indicates that these statistics could always be utilized to identify stable genotypes depending upon the experiment’s nature, breeding material, and the data complexity^63^. Regarding the correlation among stability parameters using PCA, our findings agree with Baraki et al. (2024)^35^, Benkadja et al. (2022)^65^, El-Hashash et al. (2019)^44^, Guendouz and Bendada (2022)^66^, and Pour-Aboughadareh et al. (2023)^67^ where the measures of crop yield stability are divided into different groups.

The heatmap analysis divided both genotypes and stability statistics into four clusters. The tree diagram shows the minimum distance or dissimilarity between the stability parameters inside each cluster, the opposite is true among the clusters. While genotypes statistics between the clusters differ and have the largest genetic distance, genotypes inside the cluster have the least variance and genetic distance^68^. Similar results were observed in many studies for example Al-Ashkar et al. (2019^69^; 2021^70^); Guendouz and Hafsi (2017)^71^, Pour-Aboughadareh et al. (2019)^72^, Kumbhalkar et al. (2021)^73^, and Verma et al. (2019)^74^. The stability statistics were related to the dynamic (agronomic) concept of stability, because these statistics would be desirable to choice high-yielding and stable genotypes^44^. Therefore, from our results, can select superior and stable genotypes by these stability statistics as the best statistics in NIC and DSC. Every cluster’s stability statistics had a positive linear correlation with one another, showing that they might be grouped together according to how stable every genotype is across NIC and DSC. Therefore, just one of these statistics is necessary for choosing stable genotypes in each cluster. The fact that not all of these stability parameters in clusters II, III, and IV were significantly associated with average yield suggests that they offer data that cannot be obtained solely from mean yield across NIC and DSC. According to Kilic (2012)^75^, this considerable positive association among stability statistics indicates which these indices could be utilized to identify genotypes that are both stable and well-adapted. During the relationships between correlation analysis, heatmaps, PCA, and stability parameters, the results indicated a perfect correlation among some stability statistics and highly significant and positive associations among *Yi*, *YSi*, and *TOP*, as well as the selection of the genotypes G1, G5, G19, and G20 as more stable and adaptable genotypes under drought conditions.

Finally, because of the substantial correlation between *YSi* and *TOP* with the highest production, as well as between other stability parameters evaluated with moderate or low productivity, statistics of *YSi* and *TOP* play significant and comparable roles in the stability ranking of cotton genotypes. Therefore, any one of them can be used to choose cotton genotypes that are stable and yield a lot of grain. The parametric and non-parametric stability parameters determined that genotypes G1, G5, G20, and G19 were the most dynamically stable with the high seed cotton productivity, stability, and adaptability. In contrast, the genotypes G8 and G4 were the most stable with moderate seed cotton yield across NIC and DSC. Therefore, these genotypes are utilized in cotton breeding programs to develop improved cotton varieties, ensuring sustainable production in Egypt under drought conditions.

## Conclusions

A considerable degree of genetic diversity exists among 24 genotypes for seed cotton yield across NIC and DSC, as shown by the findings of the ANOVA and AMMI analysis, which represent the varied weather conditions over the two growing seasons. Yield and performance stability should be considered concurrently to maximize the benefits of GEI and enhance the precision and improvement of genotype selection. A close relationship was observed among the parameters *Y*_*i*_, *YS*_*i*_ and *TOP* (dynamic stability). While *Y*_*i*_ has a negative correlation with the other stability statistics, which is related to the idea of static stability. To select stable and high-yielding cotton genotypes, one can use the statistics of *YSi* and *TOP*. G5 and G8 genotypes can be recommended as the most stable genotypes with the highest and moderate cotton productivity under drought-stress environments, respectively. Therefore, these genotypes are utilized in cotton breeding programs to develop improved cotton varieties that address drought conditions in Egypt.

## Supplementary Information

Supplementary Information.

## Data Availability

The data are presented within the manuscript as tables and figures. The data presented in this study are available on request from the corresponding authors.

## References

[1] Mahmood, T. et al. Genetic Potential and Inheritance Pattern of Phenological Growth and Drought Tolerance in Cotton (Gossypium Hirsutum L.). *Front. Plant Sci.* **12**, 705392 (2021). 34630456 10.3389/fpls.2021.705392 PMC8497812

[2] Abdelraheem, A., Esmaeili, N., O’Connell, M. & Zhang, J. Progress and perspective on drought and salt stress tolerance in cotton. *Ind. Crops Prod.* **130**, 118–129 (2019).

[3] El-Hashash, E. F. & Agwa, A. M. Comparison of parametric stability statistics for grain yield in barley under different drought stress severities Merit Res. *J. Agric. Sci. Soil Sci.* **6**(7), 098–111 (2018).

[4] Solis, J. et al. Genetic mapping of quantitative trait loci for grain yield under drought in rice under controlled greenhouse conditions. *Front. Chem.* **5**, 1–12. 10.3389/fchem.2017.00129 (2018). 10.3389/fchem.2017.00129 PMC5766644 29359127

[5] Yehia, W. M. B. & El-Hashash, E. F. Lint yield stability of different cotton (Gossypium barbadense L.) genotypes using GGE Biplot under normal and drought irrigation conditions. *J. Plant Product* **13**(5), 175–182 (2022).

[6] Ebem, E. C., Afuape, S. O., Chukwu, S. C. & Ubi, B. E. Genotype × environment interaction and stability analysis for root yield in sweet potato [Ipomoea batatas (L.) Lam]. *Front. Agron.* **3**, 665564. 10.3389/fagro.2021.665564 (2021).

[7] Kang, M. S. A rank–summethod for selecting high-yielding, stable corn genotypes. *Cereal Res. Comm.* **16**(1/2), 113–115 (1988).

[8] Ebdon, J. S. & Gauch, H. G. Additive main effects and multiplicative interaction analysis of national turfgrass performance trials. *Crop Sci.* **42**, 497–506 (2002).

[9] Mohammadi, R. & Amri, A. Comparison of parametric and non-parametric methods for selecting stable and adapted durum wheat genotypes in variable environments. *Euphytica* **159**, 419–432. 10.1007/s10681-007-9600-6 (2008).

[10] Sabri, R. S. et al. Assessment of agro-morphologic performance, genetic parameters and clustering pattern of newly developed blast resistant rice lines tested in four environments. *Agronomy* **10**(8), 1098. 10.3390/agronomy10081098 (2020).

[11] Yan, W. & Hunt, L. A. Interpretation of genotype × environment interaction for winter wheat yield in Ontario. *Crop Sci.* **41**(1), 19–25 (2001).

[12] Ssemakula, G. & Dixon, A. Genotype × environment interaction, stability and agronomic performance of carotenoid-rich cassava clones. *Sci. Res. Essays.* **2**, 390–399 (2007).

[13] Becker, H. C. Correlations among some statistical measures of phenotypic stability. *Euphytica* **30**, 835–840 (1981).

[14] Becker, H. C. & Leon, J. Stability analysis in plant breeding. *Plant Breed.* **101**(1), 1–23 (1988).

[15] Huehn, M. Non-parametric analysis of genotype x environment interactions by ranks. In *Genotype by environment interaction* (eds Kang, M. S. & Gauch, H. G.) 213–228 (CRC Press, 1996).

[16] Romagosa, I. & Fox, P. N. Genotype environment interaction and adaptation. (Eds.: M.D. Hayward, N.O. Bosemark and I. Romagosa), In: Plant Breeding. Principal s and Prospects, Chapman and Hall, London, 373–390 (1993)

[17] Flores, F., Moreno, M. & Cubero, J. A comparison of univariate and multivariate methods to analyze G×E interaction. *Field Crops Res.* **56**(3), 271–286. 10.1016/S0378-4290(97)00095-6 (1998).

[18] Francis, T. R. & Kannenberg, L. W. Yield stability studies in short-season maize I. A descriptive method for grouping genotypes. *Canadian J. of Plant Sci.* **58**, 1029–1034 (1978).

[19] Eberhart, S. A. & Russel, W. A. Stability parameters for comparing varieties. *Crop Sci.* **6**(1), 36–40 (1966).

[20] Wricke’s, G. Uber eine methode zur refassung der okologischen streubretite in feldversuchen. *Flazenzuecht* **47**, 92–96 (1962).

[21] Hernandez, C. M., Crossa, J. & Castillo, A. The area under the function: an index for selecting desirable genotypes. *Theo. Appl. Genet.* **87**, 409–415 (1993). 10.1007/BF00215085 24190312

[22] Shukla’s, G. K. Some aspects of partitioning genotype×enviromental components of variability. *Heredity* **28**, 237–245 (1972). 10.1038/hdy.1972.87 4507945

[23] Nassar, R. & Huehn, M. Studies on estimation of phenotypic stability: tests of significance for nonparametric measures of phenotypic stability. *Biometrics* **43**, 45–53 (1987).

[24] Fox, P. N. et al. Yield and adaptation of hexaploid spring triticale. *Euphytica* **47**, 57–64 (1990).

[25] Thennarasu’s, K. On certain non-parametric procedures for studying genotype-environment interactions and yield stability. Ph.D. Theses P.G. School IARI, New Dehli (1995).

[26] Gauch, H. G. Model selection and validation for yield trials with interaction. *Biometrics* **44**(3), 705–715. 10.2307/2531585 (1988).

[27] Suwarno, W. B. et al. PBSTAT-GE: Genotype-by-environment interaction and stability analysis for plant breeding (Version 3.6.2) [Computer software]. http://www.pbstat.com (IPB University, 2025).

[28] Allard, R. W. & Bradshaw, A. D. Implications of Genotype-Environmental Interactions in Applied Plant Breeding. *Crop Sci.* **4**(5), 503–508 (1964).

[29] Yehia, W. M. B. Evaluation of same Egyptian cotton (Gossypium barabadense L.) Genotype to water stress by using drought tolerance indices. *Elixir Agric.* **143**, 54133–54141 (2020).

[30] Sun, F. et al. Screening of Key Drought Tolerance Indices for Cotton at the Flowering and Boll Setting Stage Using the Dimension Reduction Method. *Front. Plant Sci.* **12**, 619926 (2021). 34305956 10.3389/fpls.2021.619926 PMC8299416

[31] Abo Sen, EAF et al. (2022). Evaluation of genetic behavior of some Egyptian cotton genotypes for tolerance to water stress conditions* J. of Biological Sci.* **29**(3): 1611–1617 10.1016/j.sjbs.2021.11.001 PMC8913392 35280572

[32] Rizwan, M. et al. Yield and related components of cotton (Gossypium hirsutum L.) effected by chlorophyll contents. *Pakistan J. of Agric. Res.* **35**(1), 29–35 (2022).

[33] Yehia, W. M. B., Zaazaa, E. E. D. I., El-Hashash, E. F., Abou El-Enin, M. M. & Shaaban, A. Genotype-by-environment interaction analysis for cotton seed yield using various biometrical methods under irrigation regimes in a semi-arid region. *Arch. Agron. Soil Sci.* **70**(1), 1–23. 10.1080/03650340.2023.2287759 (2023).

[34] Deshmukh, D. et al. (2025). Genotype x environment interaction analysis for fiber quality, seed cotton yield and it’s contributing traits in Bt bgii cotton (*Gosspium hirsutum* L) *Plant Arch.* **25**(1), 2300–2309.

[35] Baraki, F. et al. Parametric and Non-Parametric Measures to Identify Stable and Adaptable Cotton (*Gossypium hirsutum* L.) Genotypes. *J. Natu Fibers* **21**(1). (2024).

[36] Yehia, W. M. B. & El-Hashash, E. F. Correlation and multivariate analysis across non-segregation and segregation generations in two cotton crosses. *Egyptian J. of Agric. Res.* **99**(3), 354–364 (2021).

[37] Hashash, E. F. & Yehia, W. M. B. Estimation of heritability, genes number and multivariate analysis using non segregation and segregation generations in two cotton crosses. *Asian J. of Biochem. Genet Mol. Biol.* **9**(3), 45–62 (2021).

[38] Li, B. et al. Phenotypic plasticity and genetic variation of cotton yield and its related traits under water-limited conditions. *The Crop J.* **8**(6), 966–976 (2020).

[39] Iqbal, M. S. et al. Genotype × environment interaction analysis for yield stability of hybrid cotton across production environments through multiple biometrical tools. *J. Nat Fibers* **19**(17), 15310–15326 (2022).

[40] Lingaiah, N. et al. AMMI Biplot Analysis in Cotton (Gossypium hirsutum L.) Genotypes for genotype x environment interaction at four agro-ecologies in Telangana State. *Current J. Appl. Sci. Technol.* **39**(15), 98–103 (2020).

[41] Maleia, M. P., Jamal, E. C., Savanguane, J. W., João, J. & Teca, J. O. Stability and adaptability of cotton (Gossypium Hirsutum L.) genotypes under multi environmental conditions in mozambique. *J. of Agron. Agric. Sci.* **2**, 017 (2019).

[42] Riaz, M. et al. Stability analysis of different cotton genotypes under normal and water-deficit conditions. *J. of Integ. Agric.* **18**(6), 1257–1265 (2019).

[43] Yue, H. et al. Assessing the role of genotype by environment interaction of winter wheat cultivars using envirotyping techniques in North China. *Front. Plant Sci.* **16**, 1538661. 10.3389/fpls.2025.1538661 (2025). 40007965 10.3389/fpls.2025.1538661 PMC11850365

[44] El-Hashash, E. F. et al. Comparison of non-parametric stability statistics for selecting stable and adapted soybean genotypes under different environments Asian. *J. Res. Crop Sci.* **4**(4), 1–16 (2019).

[45] AbdElgalil, M., Mohamed, A., Gebreel, M. & Hefzy, M. Evaluation of maize varieties under conditions of water shortage in calcareous soil. *Arch. Agric. Sci. J.* **8**(3), 40–52. 10.21608/aasj.2025.468121 (2025).

[46] Ayele, A. G. et al. Responses of Upland Cotton (Gossypium hirsutum L.) Lines to Irrigated and Rainfed Conditions of Texas High Plains. *Plants* **9**, 1598 (2020). 33217966 10.3390/plants9111598 PMC7698729

[47] Eid, M. A. M. et al. Response in physiological traits and antioxidant capacity of two cotton cultivars under water limitations. *Agronomy* **12**, 803 (2022).

[48] Eissa, A., Elsaid, R., Morad, S. & Darwish, S. Evaluation of some rice (*Oryza sativa* L.) genotypes under drought stress conditions by using morphological, physiological and molecular characteristics. *Al-Azhar J. of Agric. Res.* **48**(2), 179–193. 10.21608/ajar.2023.194376.1113 (20230.

[49] Hawash, A. A. Estimation of combining ability and gene action for yield characteristics in rice under water-stress conditions. *Al-Azhar J. of Agric. Res.* **47**(2), 143–158. 10.21608/ajar.2022.277849 (2022).

[50] Thillainathan, M. & Fernandez, G. C. J. A novel approach to plant genotypic classification in multi-site evaluation. *HortSci.* **37**(5), 793–798 (2002).

[51] Marco, M., Blessing, C., Washington, M. & Dumisani, K. Exploring superiority of different cotton (Gossypium hirsutum. L.) genotypes through the application of parametric stability models. *J. Plant Sci.* **10**(4), 130–138 (2022).

[52] Murthy, K. G. K. & Pradeep, T. Study on stability parameters for yield and compact plant type characters in hybrids derived from multiple cross derivatives of upland cotton (Gossypium hirsutum L.) and amenable for HDPS. *Int J. Environ. Clim. Chan.* **12**(11), 168–179 (2022).

[53] Sirisha, A. B. M., Lal Ahamed, M., Ramakumar, P. V., Ratnakumari, S. & Srinivasa, R. V. V. Stability analysis for seed cotton yield and its component traits in upland cotton (Gossypium hirsutum L.). *Sci. Spec.* **4**(1–2), 18–27 (2019).

[54] Vavdiya, P. A., Chovatia, V. P., Bhut, N. M. & Vadodariya, G. D. G x E interactions and stability analysis for seed cotton yield and its components in cotton (Gossypium hirsutum L.). *Elect J. Plant Breed.* **12**(2), 396–402 (2021).

[55] Segherloo, A. E., Sabaghpour, S. H., Dehghani, H. & Kamrani, M. Non-parametric measures of phenotypic stability in chickpea genotypes (Cicer arietinum L.). *Euphytica* **162**, 221–229 (2008).

[56] Barroso, L. M. A. et al. Analysis of the adaptability of black bean cultivars by means of quantile regression. *Ciência Rural* **49**, e20180045 (2019).

[57] Nascimento, M. et al. Influential Points in Adaptability and Stability Methods Based on Regression Models in Cotton Genotypes. *Agronomy* **11**, 2179 (2021).

[58] Sadabadi, M. F., Ranjbar, G. A., Zangi, M. R., Tabar, S. K. K. & Zarini, H. N. Analysis of stability and adaptation of cotton genotypes using gge biplot method. *Trakia J. of Sci.* **1**, 51–61 (2018).

[59] Herawati, R., Lestari, A. P., Nurmegawati, G. D. W. & Romeida, A. Comparative study on the stability and adaptability of different models to develop a high-yield inbred line from landrace rice varieties. *Ann. Agric. Sci.* **66**(2), 184–192 (2021).

[60] Al-Ashkar, I. et al. Detection of high-performance wheat genotypes and genetic stability to determine complex interplay between genotypes and environments. *Agronomy* **13**(2), 585. 10.3390/agronomy13020585 (2023).

[61] Shojaei, S. H. et al. Evaluation of stability in maize hybrids using univariate parametric methods. *J. Crop Sci. Biotech.* **25**, 269–276 (2022).

[62] Lin, C. S., Binns, M. R. & Lefkovitch, L. P. Stability Analysis: Where Do We Stand?. *Crop Sci.* **26**(5), 894–900 (1986).

[63] Khan, M. A. U. et al. Comparison among different stability models for yield in bread wheat. *Sarhad J. Agric.* **36**(1), 282–290 (2020).

[64] Mekbib, F. Simultaneous selection for high yield and stability in common bean (*Phaseolus vulgaris*) genotypes. *The J. Agric. Sci.* **138**(3), 249–253 (2002).

[65] Benkadja, S., Maamri, K., Guendouz, A., Oulmi, A. & Frih, B. Stability analysis for grain yield and thousand kernel weight of durum wheat (Triticum durum Desf.) genotypes growing under semi-arid conditions. *Agric. Sci. Technol.* **14**(2), 34–43 (2022).

[66] Guendouz, A. & Bendada, H. Stability analysis for the grain yield of some barley (Hordeum vulgare L.) genotypes growing under semi-arid conditions. *Int. J. Bio-Resource and Stress Manag.* **13**(2), 172–178 (2022).

[67] Pour-Aboughadareh, A. et al. Deciphering Genotype-By-Environment Interaction in Barley Genotypes Using Different Adaptability and Stability Methods. *J. Crop Sci. Biotech.* **26**(5), 1–16 (2023).

[68] El-Hashash, E. F. & EL-Agoury, R. Y. Comparison of grain yield-based drought tolerance indices under normal and stress conditions of rice in Schol. *J. Agric. Vet. Sci.* **6**, 41–54 (2019).

[69] Al-Ashkar, I. et al. Detecting Salt Tolerance in Doubled Haploid Wheat Lines. *Agronomy* **9**(4), 211. 10.3390/agronomy9040211 (2019).

[70] Al-Ashkar, I. et al. Combining Genetic and Multidimensional Analyses to Identify Interpretive Traits Related to Water Shortage Tolerance as an Indirect Selection Tool for Detecting Genotypes of Drought Tolerance in Wheat Breeding. *Plants* **10**(5), 931. 10.3390/plants10050931 (2021). 34066929 10.3390/plants10050931 PMC8148561

[71] Guendouz, A. & Hafsi, M. Comparison of parametric and non-parametric methods for selecting stable and adapted durum wheat cultivars under semi-arid conditions. *Jordan J. Agric. Sci.* **13**(3), 655–662 (2017).

[72] Pour-Aboughadareh, A., Yousefian, M., Moradkhani, H., Poczai, P. & Siddique, K. H. M. STABILITYSOFT: A new online program to calculate parametric and non-parametric stability statistics for crop traits. *Appl. Plant Sci.* **7**(1), e1211 (2019). 10.1002/aps3.1211 PMC6342234 30693157

[73] Kumbhalkar, H. B., Gawande, V. L., Deshmukh, S. B., Gotmare, V. & Waghmare, V. N. Genotype x Environment interaction for seed cotton yield and component traits in upland cotton (Gossypium hirsutum L.). *Elect J. of Plant Breed.* **12**(4), 1209–1217 (2021).

[74] Verma, A., Kumar, V., Kharab, A. & Singh, G. Quantification of G×E interaction for feed barley genotypes by parametric and non-parametric measures. *Bangladesh J. Bot.* **48**(1), 33–42 (2019).

[75] Kilic, H. Assessment of parametric and nonparametric methods for selecting stable and adapted spring bread wheat genotypes in multi-environment. *J. Animal and Plant Sci.* **22**(2), 390–398 (2012).

