# Supplementary material for: Genotype by environment interaction analysis for seed cotton yield stability under normal irrigation and drought stress conditions using numerical stability statistics
Source: Sci Rep. 2026 Feb 9;16:5948. doi: 10.1038/s41598-025-33327-6 (PMC12894860; doi:10.1038/s41598-025-33327-6)
Supplement: Supplementary file 1 — Supplementary Information. [file 41598_2025_33327_MOESM1_ESM.docx]

**Data availability statement**

**Dear: Yagnasri Padma**

**Assistant Editor, Scientific Reports**

On behalf of the authors, the data presented in this study are available on request from the corresponding authors.

Your consideration of the manuscript would be greatly appreciated.

Sincerely yours,

Dr. Essam El-Hashash

Professor of Plant Breeding, Department of Agronomy, Faculty of Agricultural, Al-Azhar University, Cairo, Egypt.

Email address: dressamelhashash@azhar.edu.eg
